# A Rare Presentation of Checkpoint Inhibitor Induced Distal RTA

**DOI:** 10.1155/2021/7406911

**Published:** 2021-07-15

**Authors:** Andrew V. Doodnauth, Miriam M. Klar, Zohra R. Malik, Krunal H. Patel, Samy I. McFarlane

**Affiliations:** ^1^Department of Internal Medicine, State University of New York—Downstate Health Sciences University, Brooklyn, New York, USA 11203; ^2^Department of Internal Medicine, St. John's Episcopal Hospital, Far Rockaway, New York, USA 11691

## Abstract

Immune checkpoint inhibitors have opened a new era in treating advanced malignancies, resulting in a rapid increase in utilization, given the remarkable clinical outcomes. The incidence of immune-related adverse events increased due to the immunologic effects of these therapeutic agents. However, immune-related renal adverse events remain low, representing only a small incidence of reported cases. Common renal toxicity described includes acute interstitial nephritis, minimal change disease, and immune complex glomerulonephritis. Renal tubular acidosis has occasionally been reported but is highly uncommon. This report presents a case of a 68-year-old woman with a known history of metastatic melanoma undergoing treatment with ipilimumab+nivolumab, who developed distal renal tubular acidosis requiring stress dose steroids and sodium bicarbonate for treatment. We describe the clinical characteristics, potential mechanisms, and management of this case, highlighting the need among clinicians utilizing immune check inhibitors to be aware of this immune-related disease entity.

## 1. Introduction

Novel therapeutic agents targeting programmed cell death protein 1/programmed cell death ligand 1 (PD-1/PD-L1) and cytotoxic T-lymphocyte antigen 4 (CTLA-4) signaling are increasing in popularity. The U.S. Food and Drug Administration has approved ipilimumab and nivolumab to treat melanoma and show high rates of durable clinical responses [1, 2].

Widespread use has resulted in an increased incidence of immune-related adverse events (irAE). Recent literature has demonstrated that immune checkpoint inhibitors (ICI) may profoundly affect multiple organ systems, including the gastrointestinal tract, integumentary, pulmonary, and endocrine systems. Renal toxicity secondary to ICI therapy is associated with acute kidney injury via acute interstitial nephritis, minimal change disease, and immune complex glomerulonephritis [2]. However, the development of distal type-1 renal tubular acidosis (RTA) is rare.

Type-1 RTA is an uncommon disorder, particularly in adults, resulting in impaired distal acidification. Classic causes of new-onset distal type-1 RTA are autoimmune diseases and hypercalciuria. To date, there have only been a few reported cases of RTA associated with ipilimumab+nivolumab for the treatment of melanoma. This report describes a case of irAE inducing a distal (type-1) RTA of an elderly female. We explore and investigate ICI-induced RTA and its impact if unrecognized early.

## 2. Case Description

### 2.1. Patient Information

A 68-year-old woman with a past medical history of metastatic melanoma, hypertension, deep vein thrombosis, and pulmonary embolism with inferior vena cava filter placement presented with altered mental status, hypotension, and fever. She had received her second dose of immunotherapy with ipilimumab+nivolumab shortly before presentation.

### 2.2. Clinical Findings

Initial vitals were significant for blood pressure of 75/40 mm Hg, heart rate of 110 beats per minute, respiratory rate 24 breaths per minute, oxygen saturation 95% on room air, and temperature 98.4 F. Physical exam was remarkable for altered mental status, moderate respiratory distress, and bilateral lower extremity edema. Initial blood work before fluid resuscitation showed Hg 10.3 g/dL; WBC 13.9 K/mcL; Platelets 164 K/mcL; Na 133 mEq/L; K 3.1 mEq/L; Cl 110 mEq/L; HCO_3_^−1^ mEq/L; BUN of 23 mg/dL; Cr 3.2 mg/dL; Plasma Glucose 103 mg/dL; Total Protein 5.0 g/dL; Albumin 2.6 g/dL; Aspartate Aminotransferase (AST) 737 U/L; Alanine Aminotransferase (ALA) 399 U/L; Alkaline Phosphatase (ALK) 99 U/L; Total Bilirubin 0.6 mg/dL; Direct Bilirubin 0.4 mg/dL; Calcium 7.3 mg/dL; Magnesium1.5 mg/dL; Inorganic Phosphorus 2.5 mg/dL; Procalcitonin 59.11 ng/mL; Lactic Acid 1.7 mmol/L; PT 17.7 SEC/INR 1.46; aPTT 49.2 SEC; COVID-19 PCR negative; SARS-CoV-2-IgG negative; Influenza Type A negative; Influenza Type B negative; Venous blood gas: pH 7.24, pCO_2_ of 24 mmHg (Table 1).

Additional diagnostic evaluations included the following:

1. Electrocardiogram: normal sinus rhythm with premature ventricular contractions, heart rate of 94 beats per minute
2. CXR: no focal consolidation or pleural effusion
3. Computed tomography abdomen/pelvis w/o contrast: perinephric stranding and cholestasis
4. Computed tomography head w/o contrast: no new acute intracranial abnormality or mass effect
5. UA w/electrolytes: few white blood cells and red blood cells but no casts, urinary pH of 7.5, urine anion gap of 40, and fractional excretion of sodium of 0.6%

### 2.3. Timeline

The patient underwent prophylactic right femur nailing with excision of a metastatic lesion and developed a fever of unknown origin, hypotension, acute blood loss, and acute tubular necrosis two weeks before presentation. At that time, she completed treatment with vancomycin, aztreonam, and metronidazole due to a penicillin allergy. However, the infectious workup remained negative throughout the admission. The patient remained afebrile, hemodynamically stable, and safely discharged to a subacute rehabilitation facility.

Four days after discharge, she presented to our emergency department for fever and hypotension. The team initiated the sepsis protocol, and the patient was adequately fluid resuscitated with an appropriate blood pressure response. Blood cultures were collected, and the patient received empiric antibiotic treatment and stress dose hydrocortisone with improvement in her mental status.

Throughout the hospital course, her functional status improved significantly. The patient again agreed to placement in a subacute rehab facility on day fourteen of hospitalization.

### 2.4. Therapeautic Intervention

The clinical picture was suggestive of sepsis with multiorgan failure in the setting of a new RTA. The patient continued on antibiotics, maintenance fluids with lactated ringers, started on a sodium bicarbonate drip, and was admitted to the medical oncology service for further management. Working differential diagnoses included sepsis, adrenal insufficiency, antimicrobial toxicity, and immunotherapy adverse effects.

The patient completed a seven-day course of antibiotics. Infectious workup, including computed tomography abdominal and pelvis w/o contrast, urine culture, blood cultures, chest x-ray, and transthoracic echocardiogram (TTE), was unremarkable. While tapering the stress dose hydrocortisone, the patient developed transient hypotension, fever, and transaminitis episodes. Per discussion with the infectious disease team and primary oncologist, the thought was that the clinical presentation was likely related to immunotherapy.

Despite the acute renal failure resolution, the patient remained with a severe hyperchloremic non-anion gap metabolic acidosis (HCO_3_^−1^, the nadir of 9 mmol/L), along with persistent hypokalemia (the nadir of 2.4 mEq/L) indicating a distal (type-I) RTA.

Although possible, antimicrobial-induced RTA was unlikely due to the resolution of the renal failure and that none of the agents are known culprits to manifest acid/base disturbances [3]. Giving rise to a suspected immunotherapy-induced RTA, the patient was started on prednisone one mg/kg and transitioned to sodium bicarbonate tablets. Repeat blood work revealed an appropriate response with downtrending liver enzymes and increased serum sodium bicarbonate, indicating a resolving RTA.

Further workup revealed a negative autoimmune panel (anti-Ro (SS-A), anti-La (SS-B), ANA). SPEP, UPEP, free light chains, hepatitis serology, and thyroid function tests were unremarkable. Alkaline urine, a positive urinary anion gap, a nonanion gap metabolic acidosis with low serum bicarbonate, and persistent hypokalemia suggested a distal-type-1 RTA.

### 2.5. Follow-Up and Outcomes

The patient followed up with nephrology and oncology as an outpatient. She completed a prednisone taper over four weeks and denied symptoms at the follow-up encounter.

## 3. Discussion

The discovery of ICIs has been one of the most significant achievements in cancer treatment over the past decade, with ipilimumab, a CTLA-4 inhibitor, being the first ICI authorized for use in cancer treatment in 2011 [4]. Hodi et al. were the first to demonstrate the efficacy of ipilimumab in treating metastatic melanoma in 2010 through their phase 3 randomized, controlled trial, which compared ipilimumab plus gp100 vs. ipilimumab alone vs. gp100 alone in patients with metastatic melanoma. Demonstrating a significant increase in median overall survival in both ipilimumab groups compared to those not treated with ipilimumab [5]. The development of PD1 inhibitors (e.g., pembrolizumab and nivolumab) and PDL-1 inhibitors (e.g., atezolizumab and durvalumab) followed shortly after the CTLA-4 inhibitors and have become some of the most widely used anticancer therapies [4].

The CHECKMATE 067 trial studied nivolumab monotherapy vs. ipilimumab monotherapy vs. nivolumab plus ipilimumab combination therapy to treat patients with metastatic melanoma. Results demonstrated prolonged progression-free survival with nivolumab plus ipilimumab combination therapy (11.5 months) compared to either monotherapy group (nivolumab-6.9 months; ipilimumab-2.9 months) [6]. 3- and 5-year follow-up studies of the CHECKMATE 067 trials also demonstrated significantly increased median overall survival in patients treated with nivolumab plus ipilimumab combination therapy compared to either monotherapy group. The median overall survival for the nivolumab plus ipilimumab combination therapy group has not yet been reached at the 5-year follow-up point [7, 8]. Since then, ICIs, specifically nivolumab plus ipilimumab combination therapy, are efficacious in the treatment of renal cell carcinoma (CHECKMATE 214 trial), nonsmall cell carcinoma (CHECKMATE 227 trial), and hepatocellular carcinoma (CHECKMATE 040 trial) [9–11].

In the current care, we describe a patient with metastatic melanoma treated with nivolumab plus ipilimumab combination therapy who developed acute kidney injury (creatinine peak of 3.2) and metabolic derangements, including hypokalemia non-anion gap metabolic acidosis, suggestive of RTA.

We performed a full autoimmune workup, hepatitis, myeloma, and thyroid workup to exclude secondary causes of RTA. After excluding other medical reasons, we confidently made a diagnosis of RTA secondary to ICIs. The patient's response to steroid treatment further supports that this was an immune-related phenomenon.

Typical side effects of nivolumab and ipilimumab include those observed during the CHECKMATE 067 trial. Including diarrhea, fatigue, pyrexia, pruritus, rash, nausea, vomiting, abdominal pain, decreased appetite, arthralgia, headache, dyspnea, cough, elevated liver function tests, elevated amylase/lipase, anemia, neutropenia, pneumonitis, hypophysitis, hypo-/hyperthyroidism, colitis, and vitiligo [6, 7, 10]. No cases reported renal dysfunction during the trial or follow-up period for the CHECKMATE 067 trial. Three cases of nephritis/renal dysfunction, but not RTA, were seen in the CHECKMATE 040 trial [11].

RTA in the context of ICI immunotherapy is rare and seen in a handful of case reports. Et Bitar et al. were the first to report on a case of RTA in a patient with non-small cell lung cancer receiving treatment with nivolumab [2]. In our case, the patient presented with elevated creatinine, hypokalemia, and non-anion gap metabolic acidosis; the autoimmune workup was negative, and the patient improved with steroid treatment. Herrmann et al. presented three cases of ICI-induced RTA; of these patients, two were taking nivolumab, and one was on pembrolizumab [1]. Charmetant et al. presented a patient on both nivolumab plus pembrolizumab; their study is unique. They are the only case report to have confirmed the diagnosis with a tissue biopsy [12]. While this tissue biopsy diagnosis does not confirm that the RTA is an irAE, having a tissue diagnosis does strengthen the likelihood of the RTA being due to the ICI.

Interestingly, the patients from prior case reports had all been on nivolumab and/or pembrolizumab, both PD-inhibitors; none of these patients had been on ipilimumab. Our patient had been on both nivolumab and ipilimumab. Although a small observation sample, questioning whether RTAs are only associated with one group of ICIs (the PD-1 s) than CTLA-4 inhibitors is legitimate.

Distal (type-1) RTA is a defect in NH_4_^+^ secretion in the nephron, resulting in impaired acidification of the urine and retention of acid in the blood. Major causes in adults include autoimmune disorders, such as Sjogren's syndrome, autoimmune hepatitis, primary biliary cirrhosis, lupus, and rheumatoid arthritis; hypercalciuria including sarcoidosis and hyperparathyroidism; drug-induced, including Ifosfamide, Amphotericin B, Lithium, and Ibuprofen; and other conditions including obstructive uropathy and renal transplant rejection.

Unexplained non-anion gap metabolic acidosis in any patient should raise suspicion for RTA. Labs consistent with RTA include decreased serum bicarbonate, hypokalemia, and elevated chloride concentration. Urine pH of >5.5 in the previously listed serum abnormalities is highly suggestive of a distal RTA. Assessment of possible causes of distal RTA should include a full autoimmune workup to rule out autoimmune causes, medication reconciliation to assess for drug-induced causes, and measurement of urine calcium to assess hypercalciuria. However, physicians are to rule out other causes first to diagnose distal RTA due to ICI therapy.

Management of distal RTA secondary for ICI usage is the same as that for all autoimmune adverse events and the treatment utilized in previous cases: stress dose steroids. Treatment of hypokalemia may also be indicated depending on its severity. Prompt treatment is of utmost importance as the side effects of undiagnosed and untreated distal RTA can be drastic. These include hypokalemia and its subsequent symptoms, as well as osteoporosis due to bone resorption.

## 4. Conclusion

Immune checkpoint inhibitors have proven to be a promising approach in managing a wide array of neoplasms by immunomodulation. As these agents are becoming the standard of therapy in managing cancers, we see an increase in their use. Recent literature suggests that oncologists and nephrologists have heightened vigilance in the diagnosis of irAE, warranting attention to all adverse side effects, including renal toxicity. To our knowledge, there are only a few reported cases of ipilimumab/nivolumab-induced renal irAEs manifesting as distal RTA.

We recommend that all patients receiving checkpoint inhibitors of CTLA-4/PD-1 signaling should be monitored closely, with blood work and urine studies for early detection of renal impairment or electrolyte/acid-base abnormalities to prevent severe, irreversible renal damage.

All patients should receive corticosteroid therapy with slow taper (due to a long drug half-life), sodium bicarbonate, and the discontinuation of treatment with the CTLA-4/PD-1 immunomodulator and other nephrotoxic drugs. A multidisciplinary approach allows for a favorable prognosis.

## Figures and Tables

**Table 1:** Laboratory values during hospital admission: introduction of stress dose steroids on day seven of hospital course.

	Day 1	Day 2	Day 3	Day 4	Day 5	Day 6	Day 7	Day 8	Day 9	Day 10	Day 11	Day 12	Day 13	Day 14	References
White blood cell	13.9	10.3	14.8	11.3	8.1	11.3	11.3	10.8	12.2	14.1	13.5	13.4	16.2	16.5	3.50-10.80 K/*μ*L
Hemoglobin	10.3	8.6	9.4	8.7	8.1	8.8	8.0	8.3	9.1	9.5	9.5	9.6	10.9	10.6	14-18 g/dL
Platelet count	164	133	144	137	130	120	149	158	174	189	172	171	181	168	130-400 K/*μ*L
Serum sodium	133	141	141	144	147	149	142	145	143	142	143	142	140	141	136-145 mmol/L
Serum potassium	3.1	3.8	2.9	3.3	3.0	3.0	2.4	3.8	3.3	3.0	3.1	3.3	4.3	3.6	3.5-5.1 mmol/L
Serum chloride	110	121	117	123	125	127	120	123	120	120	120	119	118	117	98-107 mmol/L
Serum bicarbonate	11	9	13	12	12	13	16	13	15	14	14	16	16	18	21-31 mmol/L
Serum blood urea nitrogen	23	23	25	24	22	14	12	14	14	17	18	15	14	15	7-25 mg/dL
Serum creatinine	3.2	2.4	1.7	1.4	1.2	0.9	0.8	0.8	0.7	0.8	0.8	0.7	0.7	0.7	0.7-1.3 mg/dL
Serum albumin	2.6	2.6	2.8	2.8	2.9	3.0	3.0	3.1	3.1	3.2	3.0	3.3	3.2	3.3	3.4 to 5.4 g/dL
Aspartate aminotransferase	737	579	279	114	80	126	240	204	77	41	36	24	32	19	<37 U/L
Alanine aminotransferase	399	376	331	220	173	180	247	275	210	146	121	97	95	84	<55 U/L
Alkaline phosphatase	99	81	101	99	92	104	170	200	182	177	168	159	159	145	<130 U/L
Venous blood gas pH		7.24	7.31	7.28		7.31		7.34	7.34						7.32-7.43
Urine analysis pH	7.5				7.5		7.5					7.0			5.0-7.0
COVID-19 PCR	Not detected			Not detected			Not detected			Not detected			Not detected		Not detected
Influenza type A	Not detected														
Influenza type B	Not detected														

## Data Availability

The data used to support the findings of this study are included within the article.

## Additional Points

*Teaching Points.* (1) Oncologists must be aware of immune-related adverse events (irAEs) when prescribing checkpoint inhibitor therapy. (2) Unrecognized RTA can result in catastrophic long-term complications. (3) Stress dose steroids with taper, sodium bicarbonate, and discontinuation of immunomodulators are the hallmark in treating immune-related renal tubular acidosis.

## References

[1] Herrmann S. M., Alexander M. P., Romero M. F., Zand L. (2020). Renal tubular acidosis and immune checkpoint inhibitor therapy: an immune-related adverse event of PD-1 inhibitor—a report of 3 cases. *Kidney Medicine*.

[2] El Bitar S., Weerasinghe C., El-Charabaty E., Odaimi M. (2018). Renal tubular acidosis an adverse effect of PD-1 inhibitor immunotherapy. *Case Reports in Oncological Medicine*.

[3] Kim Y. W. (2007). Antimicrobial-induced electrolyte and acid-base disturbances. *Electrolyte Blood Press*.

[4] Robert C. (2020). A decade of immune-checkpoint inhibitors in cancer therapy. *Nature Communications*.

[5] Hodi F. S., O'Day S. J., McDermott D. F. (2010). Improved survival with ipilimumab in patients with metastatic melanoma. *The New England Journal of Medicine*.

[6] Larkin J., Chiarion-Sileni V., Gonzalez R. (2015). Combined nivolumab and ipilimumab or monotherapy in untreated melanoma. *The New England Journal of Medicine*.

[7] Wolchok J. D., Chiarion-Sileni V., Gonzalez R. (2017). Overall survival with combined nivolumab and ipilimumab in advanced melanoma. *The New England Journal of Medicine*.

[8] Larkin J., Chiarion-Sileni V., Gonzalez R. (2019). Five-year survival with combined nivolumab and ipilimumab in advanced melanoma. *The New England Journal of Medicine*.

[9] Motzer R. J., Tannir N. M., McDermott D. F. (2018). Nivolumab plus ipilimumab versus sunitinib in advanced renal-cell carcinoma. *The New England Journal of Medicine*.

[10] Hellmann M. D., Paz-Ares L., Bernabe Caro R. (2019). Nivolumab plus Ipilimumab in advanced non–small-cell lung cancer. *The New England Journal of Medicine*.

[11] Yau T., Kang Y. K., Kim T. Y. (2020). Efficacy and safety of nivolumab plus ipilimumab in patients with advanced hepatocellular carcinoma previously treated with Sorafenib: the Check Mate 040 Randomized Clinical Trial. *JAMA Oncology*.

[12] Charmetant X., Teuma C., Lake J., Dijoud F., Frochot V., Deeb A. (2020). A new expression of immune checkpoint inhibitors’ renal toxicity: when distal tubular acidosis precedes creatinine elevation. *Clinical Kidney Journal*.

